# Inverse Association of Serum Vitamin D in Relation to Carotid Intima-Media Thickness in Chinese Postmenopausal Women

**DOI:** 10.1371/journal.pone.0122803

**Published:** 2015-03-30

**Authors:** Yaping Hao, Xiaojing Ma, Yuqi Luo, Yiting Xu, Qin Xiong, Jiaan Zhu, Yuqian Bao, Weiping Jia

**Affiliations:** 1 Department of Endocrinology and Metabolism, Shanghai Jiao Tong University Affiliated Sixth People’s Hospital; Shanghai Clinical Center for Diabetes; Shanghai Key Clinical Center for Metabolic Disease; Shanghai Diabetes Institute; Shanghai Key Laboratory of Diabetes Mellitus, Shanghai 200233, China; 2 Department of Ultrasound, Shanghai Jiao Tong University Affiliated Sixth People’s Hospital; Shanghai Institute of Ultrasound in Medicine, Shanghai 200233, China; University of Hull, UNITED KINGDOM

## Abstract

**Objective:**

This study aimed to investigate the relationship between serum vitamin D level and carotid intima-media thickness (C-IMT) in Chinese postmenopausal women.

**Methods:**

Nine hundred and twenty six Chinese postmenopausal women without carotid artery plaque or history of cardiovascular disease were selected for analysis. Measurements of serum 25 hydroxyvitamin D_3_ (25(OH)D_3_) concentration and C-IMT were made by electrochemiluminescence immunoassay and B-mode ultrasound, respectively. Trend analysis was conducted according to tertiles of C-IMT.

**Results:**

The median serum 25(OH)D_3_ level was 11.03 ng/mL, with an interquartile range of 8.22–14.70. A decreasing trend of serum 25(OH)D_3_ level was accompanied by increased C-IMT tertiles (*P* for trend = 0.001). Correlation analysis found an inverse relationship between serum 25(OH)D_3_ level and C-IMT (*r* = –0.113, *P* = 0.001). After adjustment for confounding factors, multiple regression analysis showed that serum 25(OH)D_3_ level independently and negatively associated with C-IMT (Standard *β* = –0.112, *P* < 0.001). Moreover, the inverse correlation of serum 25(OH)D_3_ with C-IMT was also found in a subgroup of women with normal glucose tolerance, blood pressure and body mass index, and without undergoing lipid-lowering therapy (standard *β* = –0.140, *P* = 0.018).

**Conclusions:**

Serum 25(OH)D_3_ level was inversely correlated with C-IMT in Chinese postmenopausal women.

## Introduction

The incidence and risks of inadequate vitamin D in postmenopausal women have recently come to the forefront of clinicians’ attention worldwide. Studies of geographically-based prevalence of postmenopausal vitamin D inadequacy have suggested alarmingly high rates throughout European and East Asian countries [1, 2], and the rate in China is 62.8% [3]. The risks of vitamin D deficiency include metabolic disorders, which may ultimately promote development of atherosclerosis [4, 5].

Estrogen provides a protective effect against atherosclerosis; thus, the substantial depletion of estrogen following menopause puts postmenopausal women at an even higher risk of cardiovascular disease (CVD) [6, 7]. Therapeutic intervention with calcitriol can restore the impaired endothelial function caused by estrogen deficiency in rat [8], suggesting a beneficial role of vitamin D in postmenopausal subjects. Given the protective effect of vitamin D on metabolic disease and evidence that vitamin D inadequacy is prevalent in postmenopausal women, we speculate that decreased serum vitamin D level might be correlated with the indicators of atherosclerosis in postmenopausal women.

Atherosclerosis is the fundamental pathophysiology of CVD, a chronic and gradually progressive disease. CVD cases are clinically characterized as subclinical atherosclerosis or end-stage disease, the latter of which includes manifestations of coronary heart disease, ischemic heart disease, or cerebrovascular disease [9]. Progression to end-stage disease occurs over decades, and the subtle clinical signs may be overlooked if the disease is unsuspected; however, as the disease becomes more well-established, efficacy of therapeutic interventions become less robust and the lesion represents a particular challenge to completely reverse.

Early diagnosis of subclinical atherosclerosis is a key approach for preventing end-stage CVD. A useful clinical indicator of CAD risk is an increase in carotid intima-media thickness (C-IMT), detected by routine non-invasive imaging modalities [10]. For example, a 0.1 mm increment in C-IMT has been determined to increase the risk of acute myocardial infarction by 11% [11].

This cross-sectional study was designed to evaluate the relationship between C-IMT (as detected by ultrasound) and 25 hydroxyvitamin D_3_ [(25(OH)D_3_), a marker to evaluate vitamin D status] in ethnic Chinese postmenopausal women.

## Subjects and Methods

### Study subjects

The current study was conducted in accordance with the Declaration of Helsinki (1964) and with pre-approval from the Ethics Committee of Shanghai Jiao Tong University Affiliated Sixth People’s Hospital. All enrolled participants provided written informed consent.

**Fig 1:**
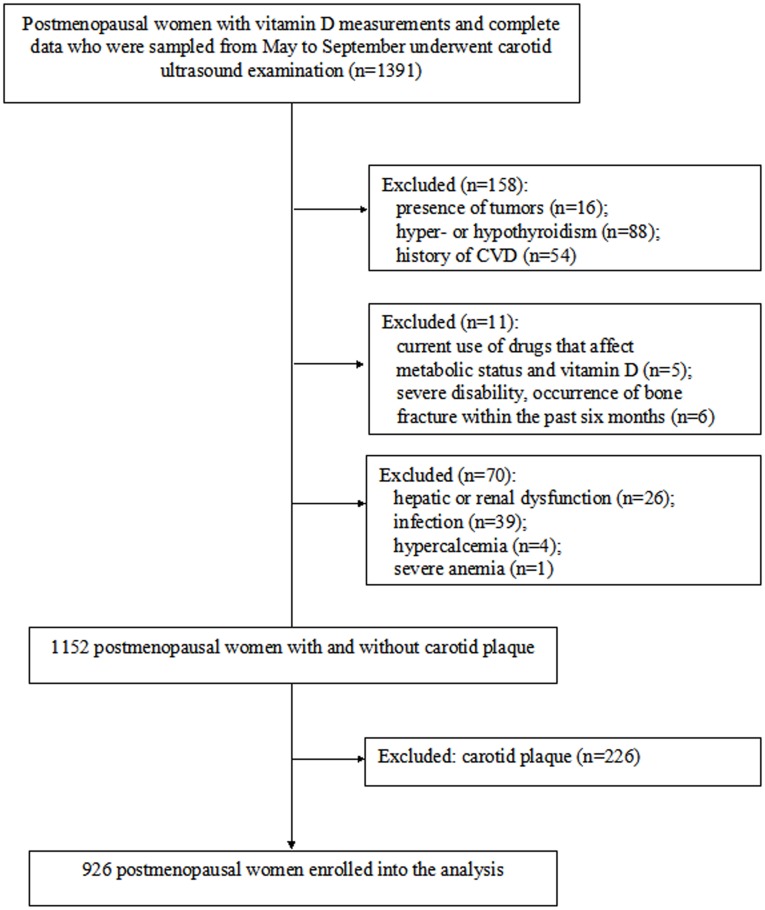
Flow chart for the selection of the study subject.

The study population enrolled in the on-going Shanghai Obesity Study (SHOS) [12] was searched for postmenopausal women with complete biological samples. A total of 1391 (age range: 45–78 years) selected postmenopausal women living in Shanghai (latitude 31° north) with complete data, vitamin D measurements and results of B ultrasonography were eligible for the present study. The study subjects were sampled from May to September which was considered as in the similar condition of sunshine, to help eliminate confounding effects of seasonal variations in sunlight exposure on serum vitamin D level. In addition, we excluded those with tumor, hyper- or hypothyroidism, history of cardiovascular disease, current use of drugs that affect metabolic status and vitamin D, severe disability or occurrence of bone fracture within the past six months, hepatic or renal dysfunction, infection, hypercalcemia [13], severe anemia, and carotid artery plaque (Fig 1). Finally, subjects from the Gonghexin, Tianmuxi and Daning communities constituted the present overall population (n = 926). Among them, 173 subjects from the Gonghexin community were enrolled during August to September in 2010, while 753 subjects from the Tianmuxi and Daning communities participated during May to September in 2011.

### Anthropometric measurements and physical activity assessment

The subject’s height (to the nearest 0.1 cm) and weight (to the nearest 0.1 kg) were used to calculate their BMI (kg/m^2^). Waist circumference (W) was measured on the midaxillary line between the inferior border of the lowest rib and the upper margin of the iliac crest. Resting blood pressure was measured in triplicate (at 3-min intervals) with a mercury column sphygmomanometer while the participants were sitting down and at rest, and reported as the averaged value.

Physical activity, a predictor of CVD and vitamin D status, was assessed as light, moderate or high according to the International Physical Activity Questionnaire (IPAQ) [14].

### Biochemical assessments

Each subject provided a fasting venous blood sample following a 10-h overnight fast. Participants without a previous history of diabetes underwent a 75 g oral glucose tolerance test, and participants with diabetes underwent a 100 g carbohydrate test. Glucose and lipid profiles were assessed by a biochemistry auto-analyzer (7600–120; Hitachi, Tokyo, Japan) as follows: fasting plasma glucose (FPG) and 2h postprandial glucose (2hPG) by glucose oxidase method; total cholesterol (TC) and triglyceride (TG) by enzymatic method; and low-density lipoprotein cholesterol (LDL-c) and high-density lipoprotein cholesterol (HDL-c) by direct assay method. Glycated hemoglobin A1c (HbA1c) was measured by high-pressure liquid chromatography (Variant II; Bio-Rad, Hercules, CA, USA). Serum fasting insulin (FINS) level was measured by electrochemiluminescence immunoassay and showed intra- and inter-assay variation coefficients of 1.7% and 2.5% respectively. The homeostasis model assessment index (HOMA-IR) was used to assess insulin resistance (IR). Serum 25(OH)D_3_ level was measured by electrochemiluminescence immunoassay (Cobas analyzer; Roche Diagnostics GmbH, Mannheim, Germany) and showed intra- and inter-assay variation coefficients of 5.6% and 8.0% respectively, with the lowest limit of sensitivity being 4 ng/mL. CRP was determined by a particle-enhanced immunonephelometry analyzer (Siemens Healthcare Diagnostic Inc., Newark, NJ).

### C-IMT measurements

A single, trained sonographer who was blinded to the clinical characteristics of participants performed the scan using the Voluson 730 Expert B-Mode Ultrasonogram with a 10-MHz probe (GE Healthcare, Waukesha, WI, USA). Both common carotid arteries were scanned proximally to the bifurcation and then distally to the bifurcation. C-IMT measurements were made at the far wall of the left and right common carotid arteries, approximately 1 cm proximal to the carotid bulb. The mean value of the maximal thickness measured for each carotid artery was calculated to give the C-IMT [15]. The intraobserver correlation between repeated C-IMT measurements was 0.962 (*P* < 0.001) [16].

### Definitions

Normal glucose tolerance (NGT) was indicated by FPG of < 6.1 mmol/L and 2hPG of < 7.8 mmol/L [17]. Normal blood pressure was defined as a systolic blood pressure (SBP) < 130 mmHg and a diastolic blood pressure (DBP) < 85 mmHg [18]. Subjects with a BMI more than 18.5 kg/m^2^ and less than 25 kg/m^2^ were classified as normal weight [19]. Current smoker status was defined as ≥ 1 cigarette/day over the past 6 months [20].

### Statistical analysis

The SPSS version 16.0 statistical software package (SPSS Inc., Chicago, IL, USA) was used for all data analyses. Data were expressed as mean ± standard deviation (for variables with normal distribution) and median with interquartile range (for variables with skewed distribution). The significance of trends was assessed by one-way ANOVA. Inter-group differences for categorical variables were assessed by the χ^2^ test. The relationships between C-IMT and clinical parameters were assessed by Spearman correlation analyses. Independent variables correlated with C-IMT, after adjustment for those correlated significantly with C-IMT in spearman correlation analysis, were identified by multiple linear regression analysis. To control for the potential confounding effects of hyperglycemia, hypertension, overweight/obesity, and lipid-lowering therapy on both C-IMT and serum 25(OH)D_3_ level in these regression analyses, a subgroup analysis was performed using only participants with relatively good health (relative to the other study participants; defined as women with glucose tolerance, blood pressure and BMI within normal ranges) and not undergoing lipid-lowering therapy. The threshold of statistical significance was set as <0.05 for two-tailed *P*-values.

## Results

### Characteristics of study participants

Table 1 summarized the demographic and clinical characteristics of the total 926 postmenopausal women classified by tertiles of C-IMT. For the total population, the median (interquartile range) level of serum 25(OH)D_3_ level was 11.03 ng/mL (8.22–14.70).

**Table 1:** Demographic and clinical characteristics of study participants.

		Tertiles of C-IMT (mm)			
Variables	Total	Q1 < 0.60 mm	Q2 0.60 –< 0.65 mm	Q3 ≥ 0.65 mm	*P* for trend
n	926	286	325	315	-
Age (year)	57.14 ± 4.62	55.90 ± 4.16	57.02 ± 4.50	58.40 ± 4.83	<0.001
BMI (kg/m^2^)	23.60 ± 3.21	23.33± 3.27	23.51 ± 3.31	23.95 ± 3.02	0.019
W (cm)	80.44 ± 8.80	79.65 ± 8.80	80.02 ± 8.72	81.59 ± 8.78	0.007
SBP (mmHg)	120.67 (111.33–130.00)	120.00 (108.67–128.67)	120.00 (112.33–130.00)	126.00 (116.67–134.67)	<0.001
DBP (mmHg)	77.33 (70.00–81.33)	73.33 (66.67–80.00)	77.33 (70.00–80.00)	79.33 (70.67–83.33)	<0.001
FPG (mmol/L)	5.29 (4.97–5.74)	5.20 (4.91–5.62)	5.32 (5.00–5.72)	5.36 (5.01–5.86)	0.008
2hPG (mmol/L)	7.06(6.07–8.67)	6.97 (5.98–8.53)	7.02 (6.11–8.57)	7.24 (6.02–8.86)	0.186
HbA1c (%)	5.70 (5.50–6.00)	5.80 (5.50–6.00)	5.70 (5.50–6.00)	5.70 (5.50–6.00)	0.395
TC (mmol/L)	5.62 ± 0.94	5.68± 0.95	5.53 ± 0.85	5.66 ± 1.02	0.844
TG (mmol/L)	1.32 (0.97–1.84)	1.24 (0.97–1.84)	1.25 (0.94–1.78)	1.42 (1.01–2.02)	0.048
HDL-c (mmol/L)	1.51 (1.30–1.75)	1.55 (1.32–1.79)	1.52 (1.28–1.76)	1.47 (1.29–1.72)	0.134
LDL-c (mmol/L)	3.42 ± 0.80	3.45 ± 0.79	3.37 ± 0.73	3.45 ± 0.87	0.993
FINS (mU/L)	7.44 (5.40–10.45)	7.06 (4.70–10.16)	7.43 (5.54–10.22)	7.75 (6.03–10.92)	0.015
HOMA-IR	1.78 (1.23–2.62)	1.67 (1.07–2.46)	1.77 (1.27–2.58)	1.86 (1.38–2.80)	0.012
CRP (mg/L)	0.81 (0.41–1.58)	0.76 (0.40–1.55)	0.77 (0.39–1.58)	0.88 (0.46–1.67)	0.486
25(OH)D_3_ (ng/mL)	11.03 (8.22–14.70)	11.97 (8.56–15.83)	10.75 (8.09–14.31)	10.63 (7.69–14.21)	0.001
Years since menopause (year)	7.33 (3.28–11.15)	5.69 (2.40–10.46)	7.31 (3.88–11.40)	8.32 (3.55–11.35)	<0.001
Physical activity (high), n(%)	452 (48.8)	142 (49.7)	158 (48.6)	152 (48.3)	0.341
Current smoker, n(%)	11 (1.2)	6 (2.1)	3 (0.9)	2 (0.6)	0.296
Antidiabetic therapy, n(%)	43 (4.6)	11 (3.8)	15 (4.6)	17 (5.4)	0.367
Anti-hypertensives, n(%)	183 (19.8)	38 (13.3)	60 (18.5)	85 (27.0)	<0.001
Lipid-lowering therapy, n(%)	32 (3.5)	1 (0.3)	10 (3.1)	21 (6.7)	<0.001
Family history of CVD, n(%)	295 (31.9)	86 (30.1)	102 (31.4)	107 (34.0)	0.303

Data were shown as mean ± SD or median (interquartile range).

Abbreviation: C-IMT, carotid intima-media thickness; BMI, body mass index; W, waist circumference; SBP, systolic blood pressure; DBP, diastolic blood pressure; FPG, fasting plasma glucose; 2hPG, 2h postprandial plasma glucose; HbA1c, glycated hemoglobin A1c; TC, total cholesterol; TG, triglyceride; HDL-c, high-density lipoprotein cholesterol; LDL-c, low-density lipoprotein cholesterol; FINS, fasting insulin; HOMA-IR, homeostasis model assessment for insulin resistance; CRP, C-reactive protein; 25(OH)D_3_, 25 hydroxyvitamin D_3_; CVD, cardiovascular disease.

Analysis of the C-IMT tertiles showed that increases in C-IMT were accompanied by increased age, BMI, W, blood pressure, FPG, TG, FINS, HOMA-IR, years since menopause, frequency of anti-hypertensives and lipid-lowering therapy (*P* for all trends < 0.05). In addition, a marked descending trend in serum 25(OH)D_3_ level was also found to accompany the increase in C-IMT tertiles (*P* for trend = 0.001).

### Association of C-IMT with serum 25(OH)D_3_ level and related clinical parameters

As shown in Table 2, Spearman correlation analysis indicated that positive correlations existed between C-IMT and age, BMI, W, blood pressure, FPG, TG, FINS, HOMA-IR and years since menopause, frequency of anti-hypertensives and lipid-lowering therapy (all *P* < 0.05), and that a negative correlation existed between C-IMT and serum 25(OH)D_3_ level (*P* = 0.001).

**Table 2:** Spearman’s correlation analysis between C-IMT and clinical variables.

Variables	*r*	*P*
Age	0.234	<0.001
BMI	0.110	0.001
W	0.118	<0.001
SBP	0.224	<0.001
DBP	0.147	<0.001
FPG	0.093	0.004
2hPG	0.047	0.155
HbA1c	–0.008	0.817
TC	–0.006	0.854
TG	0.073	0.026
HDL-c	–0.064	0.053
LDL-c	–0.004	0.910
FINS	0.107	0.001
HOMA-IR	0.120	<0.001
CRP	0.063	0.056
25(OH)D_3_	–0.113	0.001
Years since menopause	0.151	<0.001
Physical activity	–0.003	0.926
Smoking status	–0.003	0.919
Antidiabetic therapy	0.039	0.238
Anti-hypertensives	0.147	<0.001
Lipid-lowering therapy	0.130	<0.001
Family history of CVD	0.038	0.245

Abbreviation: C-IMT, carotid intima-media thickness; BMI, body mass index; W, waist circumference; SBP, systolic blood pressure; DBP, diastolic blood pressure; FPG, fasting plasma glucose; 2hPG, 2h postprandial plasma glucose; HbA1c, glycated hemoglobin A1c; TC, total cholesterol; TG, triglyceride; HDL-c, high-density lipoprotein cholesterol; LDL-c, low-density lipoprotein cholesterol; FINS, fasting insulin; HOMA-IR, homeostasis model assessment for insulin resistance; CRP, C-reactive protein; 25(OH)D_3_, 25 hydroxyvitamin D_3_; CVD, cardiovascular disease.

Multiple linear regression analysis, with C-IMT set as the dependent variable was performed. After adjusting for age, BMI, W, SBP, DBP, FPG, TG, FINS, years since menopause, 25(OH)D_3_, anti-hypertensives and lipid-lowering therapy, we found that serum 25(OH)D_3_ level (standard *β* = –0.112; *P* < 0.001) was determined to be one of the independent factors of C-IMT, along with age, BMI, SBP, and lipid-lowering therapeutic intervention (Table 3).

**Table 3:** Independent factors of increased C-IMT identified by multiple linear regression analysis.

Independent variables	Standard *β*	*t* value	*P*
Age	0.208	6.533	<0.001
BMI	0.083	2.599	0.010
SBP	0.154	4.735	<0.001
25(OH)D_3_	–0.112	–3.610	<0.001
Lipid-lowering therapy	0.074	2.370	0.018

Variables included in the model were: age, BMI, W, SBP, DBP, FPG, TG, FINS, years since menopause, 25(OH)D_3_, anti-hypertensives and lipid-lowering therapy.

Abbreviation: C-IMT, carotid intima-media thickness; BMI, body mass index; SBP, systolic blood pressure; 25(OH)D_3_, 25 hydroxyvitamin D_3_.

Multiple linear regression analysis of the relatively healthy subgroup of women was conducted, with the C-IMT continuous variable set as the dependent variable (Table 4). Consequently, after adjustments for age, BMI, W, SBP, DBP, FPG, TG, FINS, years since menopause, 25(OH)D_3_, serum 25(OH)D_3_ level still remained as an independent variable of C-IMT (standard *β* = –0.140, *P* = 0.018).

**Table 4:** Independent factors of C-IMT identified by multiple linear regression analysis of a healthy subgroup.*

Variables Independent variables	Standard *β*	*t* value	*P*
Age	0.190	3.227	0.001
FINS	0.145	2.455	0.015
25(OH)D_3_	–0.140	–2.388	0.018

*Subjects with normal glucose tolerance, blood pressure and body mass index, and without lipid-lowering therapy.

Variables included in the model were: age, BMI, W, SBP, DBP, FPG, TG, FINS, years since menopause, 25(OH)D_3_.

Abbreviation: C-IMT, carotid intima-media thickness; FINS, fasting insulin; 25(OH)D_3_, 25 hydroxyvitamin D_3_.

## Discussion

The key finding of the current study was that postmenopausal Chinese women showed a decreasing trend in serum 25(OH)D_3_ level that accompanied increases in C-IMT. Statistical analyses of the related data suggested that serum 25(OH)D_3_ concentration was an independent factor for C-IMT in this population.

Accumulating evidence in the literature supports the theory that vitamin D deficiency is an important contributing factor to the development of atherosclerosis in postmenopausal women, yet clinical studies of the precise interrelationship of this physiological factor and disease have yielded inconsistent results. A 16-year prospective follow-up study of postmenopausal Caucasian European women indicated that the risks of CVD and related death were higher in women with vitamin D deficiency than in their counterparts with normal levels [21]. In contrast, a study of postmenopausal Caucasian American women, who were overweight/obese, showed no association between vitamin D levels and CVD risk [22]. The results from psychological, social and biological determinants of ill health (pSoBid) showed that despite the lower levels of 25(OH)D in deprived groups, there was no association of vitamin D with C-IMT [23]. The differences in geographic location (possibly highlighting some ethnicity-related genetic differences) and general health or metabolic status may explain the discrepancies.

The influential roles of ethnicity and regional-related factors on serum vitamin D levels are well established [24]. In the present study, only postmenopausal women who resided in Shanghai (latitude 31°N) were enrolled and analysis showed that serum 25(OH)D_3_ level was a negative indicator of increased C-IMT. These findings agree with the collective evidence from previous related studies, which have revealed inverse relationships between serum vitamin D level and metabolic disorders [25, 26]. Furthermore, when potentially confounding factors were excluded from the current analysis (using a subgroup of healthy participants), the inverse association of serum 25(OH)D_3_ level and C-IMT was maintained. This evidence represents the first of its kind to demonstrate the relationship between serum vitamin D level and C-IMT in relatively healthy, postmenopausal Chinese women.

The mechanisms that underlie the association of vitamin D with C-IMT in the present study may be speculated upon by considering the literature on roles of vitamin D under normal physiological conditions and in cardiovascular-related pathogeneses. Vitamin D has been shown to exert cardio-protective effects by inhibiting cholesterol uptake and the formation of foam cells. In addition, vitamin D has been shown to prevent the development of atherogenesis by suppressing cholesterol uptake and endoplasmic reticulum stress [27]. Vitamin D deficiency might activate the renin-angiotensin system, increase serum level of parathyroid hormone (PTH), and decrease insulin-like growth factor 1 (IGF-1) level. In this way, vitamin D deficiency contributes to increasing cardiovascular risk [28–30]. Further studies are warranted to investigate the pathophysiologic association of vitamin D with C-IMT.

Aging, obesity, hypertension and insulin resistance were identified as risk factors of atherosclerosis in the current study of Chinese women. These risk factors have also been identified by the American Heart Association, based upon their consideration of a broader range of ethically diverse individuals [31].

The results of the current study should be interpreted cautiously, as some limitations to the study design may impact their generalizability. First, the study population used was relatively small and consisted of only postmenopausal women from a restricted geographic region. Second, the cross-sectional design precluded the ability to determine the causal relationship between vitamin D and C-IMT. Third, PTH, IGF-1 and renin-angiotensin system were not considered. Nevertheless, the present study has two key strengths. To our knowledge, it is the first to examine C-IMT and serum vitamin D level in a population of postmenopausal Chinese women and included subgroup analysis of a relatively healthy population.

In conclusion, this study provides the first published evidence of a correlation between decreased serum vitamin D level and increased C-IMT in Chinese postmenopausal Chinese women.
